# Ultrasound-Guided Targeted Injection to the Anterior Labral–Ligamentous Complex of the Shoulder: A Cadaveric Feasibility Study for Regenerative Therapy

**DOI:** 10.3390/bioengineering13040418

**Published:** 2026-04-02

**Authors:** Sang-Hyun Kim, U-Young Lee, Yonghyun Yoon, Jihyo Hwang, Jungyoun Kim, Yoonju Na, Seungbeom Kim, King Hei Stanley Lam, Jeimylo C. de Castro, Teinny Suryadi, Anwar Suhaimi

**Affiliations:** 1 College of Korean Medicine, Woosuk University, 443, Samnye-ro, Samnye-eup, Wanju-gun, Jeonju 55338, Republic of Korea; amalang@naver.com; 2 Department of Anatomy, Catholic Institute for Applied Anatomy, College of Medicine, The Catholic University of Korea, Seoul 06591, Republic of Korea; cicloaum@catholic.ac.kr; 3 Department of Orthopaedic Surgery, Gangnam Sacred Heart Hospital, Hallym University College of Medicine, 1 Singil-ro, Yeongdeungpo-gu, Seoul 07441, Republic of Korea; hwangjihyo36@gmail.com; 4 Board of Clinical Research, The International Association of Musculoskeletal Medicine, Kowloon, Hong Kong; drlamkh@gmail.com (K.H.S.L.); painfreedoc22@gmail.com (T.S.); anwar@ummc.edu.my (A.S.); 5 MSKUS, 1035 E. Vista Way #128, Vista, CA 92084, USA; 6 International Academy of Regenerative Medicine, Inha-ro 489beon-gil, Namdong-gu, Incheon 21574, Republic of Korea; stplayer@naver.com; 7 Department of Physical Medicine and Rehabilitation, Kangbuk Samsung Hospital, Sungkyunkwan University School of Medicine, Seoul 03181, Republic of Korea; 8 Miso Pain Clinic, 1569, Bongyeong-ro, Yeongtong-gu, Suwon-si 16703, Republic of Korea; 9 SMARTMD Center for Non-Surgical Pain Interventions, Makati 1205, Philippines; jeidec@yahoo.com.ph; 10 Research Department, Adventist University of the Philippines (A.U.P.), Puting Kahoy, Silang 4118, Philippines; 11 Department of Rehabilitation Medicine, Universiti Malaya, Kuala Lumpur 50603, Malaysia

**Keywords:** anterior labral–ligamentous complex, inferior glenohumeral ligament, ultrasound-guided injection, shoulder instability, frozen shoulder, regenerative injection therapy, capsulolabral interface, cadaveric validation

## Abstract

The anterior labral–ligamentous complex (ALLC), formed by the integration of the anterior inferior glenohumeral ligament and anterior glenoid labrum, plays a critical role in shoulder stability and represents a potential target for regenerative injection therapy. However, precise ultrasound-guided targeting of this capsular–labral interface has not been anatomically validated. This feasibility cadaveric study evaluated the accuracy of ultrasound-guided in-plane injection directed at the ALLC at its glenoid attachment. A fresh-frozen human cadaver specimen was examined in the supine position with the shoulder in external rotation. Stepwise lateral-to-medial and cranial-to-caudal ultrasound scanning was performed to localize the ALLC. A 23-gauge needle was advanced in a cranial-to-caudal in-plane trajectory, and 1–2 mL of red filler was injected into the capsular–labral interface. Subsequent layer-by-layer dissection was conducted to assess injectate localization. In both shoulders (2/2), red filler was confined within the ALLC without extra-capsular leakage, diffuse intra-articular pooling, or neurovascular staining. These findings demonstrate the anatomical feasibility of ultrasound-guided targeted delivery to the anterior capsular–labral complex. This proof-of-concept study provides foundational anatomical validation for the development of regenerative injection protocols directed at the shoulder capsular–labral enthesis.

## 1. Introduction

The shoulder joint possesses the greatest range of motion in the human body; however, this mobility comes at the expense of inherent instability. Joint stability is maintained by a complex interplay of static and dynamic stabilizers, including the osseous architecture, glenoid labrum, capsuloligamentous structures, negative intra-articular pressure, and surrounding musculature.

Among these static stabilizers, the inferior glenohumeral ligament (IGHL) complex plays a pivotal biomechanical role [1]. Functioning as a hammock-like structure between the glenoid and proximal humerus, the IGHL dynamically tensions according to arm position and serves as a primary restraint against anterior and inferior translation of the humeral head [2]. The anterior bundle of the IGHL is particularly important in positions of external rotation and abduction, where it resists anterior instability [3].

At its glenoid insertion, the anterior IGHL is anatomically integrated with the anterior glenoid labrum [4]. Rather than functioning as an isolated ligament, it forms a continuous labral–ligamentous complex at the enthesis level, herein referred to as the anterior labral–ligamentous complex (ALLC) [5]. This integrated capsular–labral interface represents a unified structural and functional unit contributing to anterior shoulder stability.

Clinically, pathology involving the ALLC demonstrates age-dependent relevance. In younger individuals, injury at the labral–ligamentous attachment is closely associated with anterior shoulder instability and Bankart lesions [3,6]. In contrast, in middle-aged and older patients, capsular thickening involving anterior capsuloligamentous structures has been implicated in adhesive capsulitis, in which altered capsular compliance contributes to restricted motion and pain [7,8].

Despite its established biomechanical and surgical importance, most prior investigations have focused on arthroscopic repair techniques or biomechanical cadaveric analyses. Image-guided interventions specifically targeting the ALLC at its glenoid attachment have not been systematically evaluated. Conventional intra-articular injections deliver medication diffusely within the joint cavity and may not adequately address localized capsulolabral pathology [9,10].

With advances in high-resolution musculoskeletal ultrasound, precise visualization of capsulolabral and ligamentous structures has become increasingly feasible [11,12]. However, anatomical validation of ultrasound-guided targeted injection to the ALLC remains lacking. Previous ultrasound-guided studies have largely emphasized confirmation of intra-articular needle placement rather than structural confinement at the enthesis-level interface.

From a delivery-engineering perspective, regenerative interventions targeting capsuloligamentous pathology may require precise localization and confinement within the capsular–labral interface to maximize biological effect while minimizing dilution within the joint space. Cadaveric validation studies have been increasingly utilized to confirm anatomical accuracy in ultrasound-guided musculoskeletal procedures, as dissection-based confirmation allows direct assessment of injectate localization beyond sonographic appearance alone.

Accordingly, this feasibility cadaveric validation study aimed to determine whether an ultrasound-guided technique can achieve accurate targeted delivery and confinement of red filler to the ALLC at its glenoid attachment using an MRI-equivalent shoulder position, and to confirm localization through subsequent anatomical dissection.

## 2. Materials and Methods

### 2.1. Study Design and Specimen

This study was designed as a feasibility cadaveric validation study to determine whether an ultrasound-guided technique can achieve accurate targeted delivery of injectate to the ALLC at its glenoid attachment site.

The ALLC was defined as the integrated attachment of the anterior inferior glenohumeral ligament and the anterior glenoid labrum at the glenoid margin.

A single fresh-frozen human cadaver specimen was used. The specimen was thawed at room temperature prior to the procedure to approximate physiological tissue conditions. Each shoulder was considered an independent technical attempt using an identical standardized protocol.

### 2.2. Ultrasound Equipment and Imaging Parameters

Ultrasonographic evaluation was performed using an Alpinion XC90 Elite ultrasound system (ALPINION MEDICAL SYSTEMS Co., Ltd., Seoul, Republic of Korea) equipped with a high-frequency linear transducer.

Imaging parameters were standardized (depth: 4 cm; dynamic range: 60 dB). A lower-frequency setting (7 MHz) was selected to ensure adequate penetration for visualization of the deep capsulolabral structures. Coupling gel was applied to optimize acoustic transmission.

Dynamic scanning was performed to identify the glenoid rim, humeral head, and anterior capsulolabral structures. Particular attention was given to the ALLC at the glenoid attachment, representing the continuous integration of the anterior IGHL and anterior glenoid labrum.

### 2.3. Positioning

The cadaver was positioned supine. The shoulder was placed in external rotation to reproduce the MRI evaluation position commonly used to assess anterior capsulolabral structures.

This positioning allowed optimal visualization of the ALLC at the glenoid attachment while maintaining anatomical alignment and facilitating reproducible probe orientation.

### 2.4. Ultrasound-Guided Targeted Injection

Under real-time ultrasound guidance, a 23-gauge, 6 cm needle was advanced using an in-plane technique in a cranial-to-caudal direction to target the ALLC at its glenoid attachment site. This cranial-to-caudal trajectory was selected to optimize needle visualization and to maximize the safety margin relative to adjacent neurovascular structures. By entering superiorly and advancing toward the mid-glenoid level, the needle pathway remains outside the typical inferior neurovascular course of the axillary nerve and posterior humeral circumflex vessels, thereby reducing the risk of iatrogenic injury compared with inferior or perpendicular trajectories.

Needle advancement was performed with continuous visualization of the needle shaft and tip to ensure accurate positioning within the intended capsular–labral interface. Care was taken to maintain the needle tip within the capsule and to avoid penetration into the joint cavity, while minimizing proximity to adjacent neurovascular structures.

All injections were performed by an orthopedic surgeon (Y.Y.) with more than 10 years of experience in musculoskeletal ultrasonography. At the target site, 1–2 mL of a red-colored filler solution was injected. The injection volume was deliberately minimized to allow gross visualization during dissection while reducing the risk of unintended intra-articular spread. Needle placement and red filler deposition were monitored in real time. Procedural ultrasound images and video recordings were obtained for documentation and technical illustration.

### 2.5. Anatomical Dissection and Outcome Assessment

Following injection, anatomical dissection was performed by a professional anatomist with more than 10 years of cadaveric dissection experience.

A meticulous layer-by-layer approach was used to expose the deltoid, subscapularis, and anterior capsule while preserving capsulolabral structures.

The capsule was incised vertically at its humeral attachment and reflected medially to directly inspect the glenoid-side insertion of the ALLC. Particular attention was paid to the integrated labrum–ligament attachment at the glenoid margin to evaluate red filler localization within the capsular–labral complex.

Surrounding neurovascular structures, including the axillary nerve and posterior humeral circumflex artery, were carefully assessed to detect any unintended filler spread.

### 2.6. Outcome Definition

The primary outcome was technical success, defined as:

- Grossly visible focal localization of red filler within the ALLC inside the joint capsule
- Absence of extra-capsular spread
- Absence of diffuse intra-articular pooling within the glenohumeral joint cavity
- No staining of adjacent neurovascular structures

Diffuse intra-articular red filler accumulation was categorized as non-targeted spread.

Feasibility was assessed on a per-shoulder basis, with each shoulder considered an independent technical attempt using the same standardized protocol. Gross photographs were obtained to document filler localization patterns.

### 2.7. Ethics Statement

This study was conducted as a feasibility cadaveric validation study to determine whether an ultrasound-guided technique can achieve targeted injectate delivery to the glenoid attachment of the inferior glenohumeral ligament. The protocol was reviewed by the Institutional Review Board of the Catholic University of Korea and was granted an exemption from full ethical review because the study involved cadaveric specimens only and did not include living human participants or identifiable personal data (IRB No. MIRB-면20260120-006; 20 January 2026).

In addition, representative ultrasound images included in this manuscript were obtained from a clinical case for illustrative purposes. Written informed consent was obtained from the patient for the use of anonymized images in this publication. The images do not contain identifiable personal information.

## 3. Results

### 3.1. Ultrasound Landmark Identification and Localization of the ALLC

Ultrasound imaging enabled stepwise identification and targeted localization of the ALLC at its glenoid attachment site.

**Figure 1:**
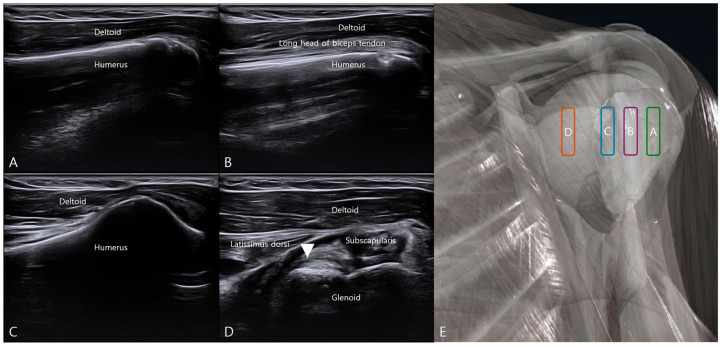
Lateral-to-medial probe translation for identification of the anterior labral–ligamentous complex. (**A**–**D**) Sequential ultrasound images obtained during stepwise lateral-to-medial probe translation in the supine position. (**A**) Lateral starting view at the level of the bicipital groove. (**B**) Centered view over the bicipital groove. (**C**) Medial translation beyond the groove. (**D**) Visualization of the glenoid rim and anterior labral–ligamentous complex. (**E**) Schematic illustration of probe positions corresponding to panels (**A**–**D**). The white triangle indicates the anterior labral–ligamentous complex.

With the cadaver in the supine position, the transducer was initially placed perpendicular to the humeral shaft to establish a reproducible lateral starting view. The probe was then translated medially in a controlled manner, maintaining a consistent orientation while progressively shifting the sonographic window from the humeral head toward the glenoid rim (Figure 1).

This lateral-to-medial scanning sequence allowed systematic identification of key osseous landmarks, including the humeral head and glenoid margin, which served as stable anatomical references for anterior capsulolabral assessment. The anterior capsule was subsequently delineated between the glenoid and humeral head, enabling differentiation of capsular tissue from adjacent musculotendinous structures.

**Figure 2:**
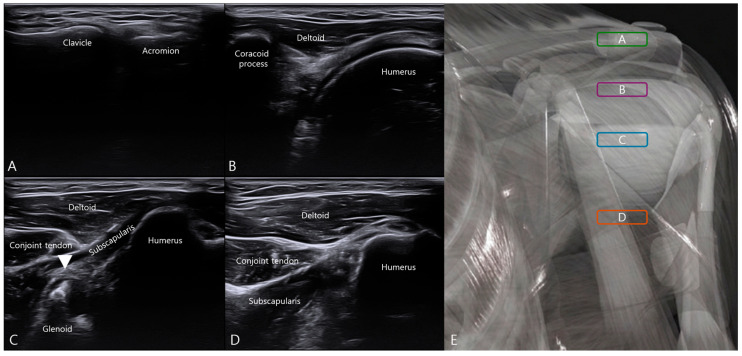
Cranial-to-caudal probe translation for vertical localization of the anterior labral–ligamentous complex. (**A**–**D**) Sequential ultrasound images obtained during cranial-to-caudal probe translation. (**A**) Level of the acromioclavicular joint. (**B**) Level of the coracoid process and proximal humerus. (**C**) Inferior to the coracoid process. (**D**) Level at which the glenoid is no longer visualized. (**E**) Schematic representation of probe position corresponding to panels (**A**–**D**). The white arrowhead indicates the anterior labral–ligamentous complex.

Continued medial translation and subtle probe adjustment permitted visualization of the integrated labrum–ligament attachment at the anterior glenoid margin, corresponding to the ALLC. Additional cranial-to-caudal probe translation was performed to confirm vertical localization of the anterior labral–ligamentous complex (Figure 2).

With subtle probe angulation and dynamic adjustment under external rotation positioning, the ALLC was visualized as a distinct capsulolabral thickening at its glenoid attachment. Fine probe adjustments permitted clear delineation of the integrated labrum–ligament interface along the anterior glenoid margin.

This sequential landmark-based approach provided a reproducible sonographic roadmap from stable osseous references to the deeper capsular–labral complex, enabling precise target localization under real-time ultrasound guidance.

### 3.2. Ultrasound-Guided In-Plane Injection

**Figure 3:**
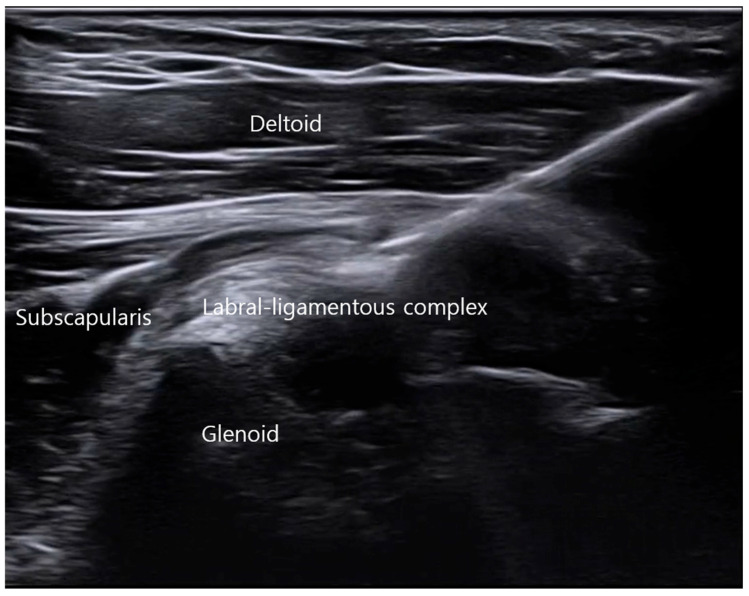
Ultrasound-guided in-plane needle approach targeting the anterior labral–ligamentous complex.

Using the above localization strategy, ultrasound-guided injection was performed with an in-plane technique. The needle was advanced in a cranial-to-caudal direction with continuous visualization of the needle shaft and tip relative to the ALLC at its glenoid attachment site (Figure 3). Repeated confirmation ensured accurate positioning within the intended capsular–labral interface while avoiding intra-articular penetration.

A small volume (1–2 mL) of red filler was delivered at the target site. Real-time monitoring demonstrated controlled deposition confined to the anterior labral–ligamentous complex, supporting the technical feasibility of in-plane access to the capsular–labral complex under ultrasound guidance.

Representative ultrasound image demonstrating an in-plane, cranial-to-caudal needle approach directed toward the anterior labral–ligamentous complex at the glenoid attachment site. The needle tip is positioned within the capsular–labral interface prior to delivery of 1–2 mL of red filler.

### 3.3. Cadaveric Dissection Confirmation of Injectate Localization (Bilateral Shoulders)

Subsequent layer-by-layer dissection demonstrated focal red filler localization at the glenoid attachment site of the anterior labral–ligamentous complex (ALLC) in both shoulders. Prior to capsular incision, no extracapsular leakage was observed through the subscapularis muscle or superficial capsule.

**Figure 4:**
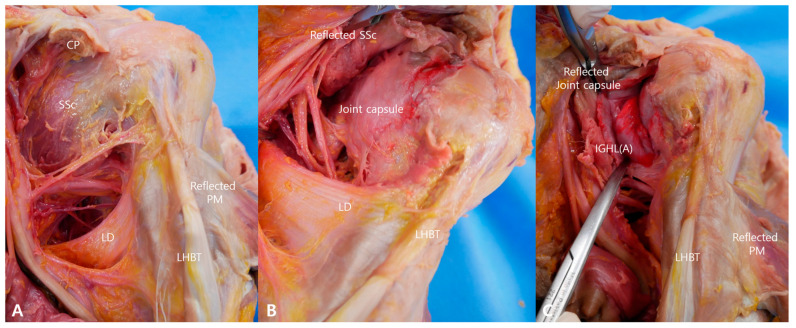
Representative cadaveric dissection demonstrating red filler localization within the anterior labral–ligamentous complex. (**A**) Reflection of the conjoint tendon at the level of the coracoid process and reflection of the pectoralis major to expose the anterior shoulder structures. (**B**) Reflection of the subscapularis muscle demonstrating intact capsule without extra-capsular red filler leakage. (**C**) Vertical capsular incision with medial reflection revealing red filler confined within the anterior labral–ligamentous complex at the glenoid attachment.

Following vertical incision of the capsule at its humeral attachment and medial reflection, red filler was directly visualized within the integrated capsular–labral complex at the anterior glenoid margin (Figure 4). The injectate was entirely confined within the targeted capsular–labral interface and did not extend beyond the capsule.

Gross inspection confirmed that red filler was deposited at the intended ALLC without diffuse distribution into the glenohumeral joint cavity, posterior compartment, or superior recesses. No staining of adjacent neurovascular structures, including the axillary nerve or posterior humeral circumflex artery, was identified, indicating that low-volume (1–2 mL) delivery can achieve focal concentration at the ALLC.

Overall, technical success was achieved bilaterally (2/2 shoulders), supporting the feasibility and within-specimen reproducibility of the ultrasound-guided in-plane targeting technique. For illustrative purposes, representative ultrasound and dissection images from one shoulder are presented; similar findings were confirmed in the contralateral shoulder.

## 4. Discussion

The present study demonstrates the anatomical feasibility of ultrasound-guided targeted injection to the glenoid attachment site of the ALLC, confirmed through cadaveric dissection. Confinement of red filler within the capsular–labral interface, without diffuse intra-articular spread, supports the technical precision of the described in-plane, cranial-to-caudal approach.

### 4.1. Biomechanical Significance of the Anterior Labral–Ligamentous Complex

The IGHL complex is widely recognized as the primary static stabilizer of the shoulder, particularly in positions of abduction and external rotation. The anterior bundle serves as a key restraint against anterior and inferior translation of the humeral head [13,14]. At its glenoid insertion, however, the anterior IGHL is structurally integrated with the anterior glenoid labrum, forming a continuous labral–ligamentous complex at the enthesis level [4,6].

This integrated capsular–labral interface functions as a unified structural unit contributing to anterior shoulder stability. Injury, attenuation, or capsular thickening involving this region has been implicated in anterior instability in younger individuals and motion restriction patterns in adhesive capsulitis among older patients. Despite this biomechanical relevance, image-guided interventions specifically targeting the ALLC have not been systematically evaluated.

### 4.2. Rationale for Interface-Level Regenerative Delivery

The significance of this study extends beyond procedural feasibility. Regenerative approaches—including prolotherapy, platelet-rich plasma (PRP), and cell-based therapies—aim to stimulate collagen remodeling and structural reinforcement at sites of ligamentous or enthesis-level pathology [15,16,17,18]. However, therapeutic efficacy may depend on accurate delivery to the pathological interface rather than diffuse distribution within the joint cavity.

Previous ultrasound-guided studies have primarily focused on confirming intra-articular needle placement [9,10]. In contrast, the present study emphasizes targeted confinement at the capsular–labral interface. From a delivery-engineering perspective, maintaining injectate concentration at a defined enthesis site may enhance local bioavailability while minimizing dilution within the glenohumeral joint space [19,20,21].

The absence of injectate leakage observed in this study suggests that focal confinement at the ALLC is technically achievable. This may have translational relevance when high-cost biologic agents are used, as minimizing intra-articular diffusion could improve delivery efficiency and reduce unnecessary volume requirements.

### 4.3. Technical Considerations

A key technical component of the procedure was the cranial-to-caudal in-plane approach with continuous needle visualization. This trajectory enabled controlled advancement toward the capsular–labral interface while reducing the likelihood of unintentional intra-articular penetration.

The cranial entry point was selected to maximize the safety margin relative to the axillary nerve and posterior humeral circumflex vessels, which course inferiorly and medially. By advancing from superior to inferior along a parallel orientation to the glenoid rim, the needle pathway remains outside the typical inferior neurovascular corridor, thereby reducing theoretical risk compared with inferior or perpendicular trajectories [22,23,24].

Additionally, the cranial-to-caudal in-plane orientation facilitated optimal needle conspicuity using dynamic heel-to-toe probe adjustments [25,26]. Alignment between the ultrasound beam and needle shaft improved echogenicity and real-time tracking of the needle tip throughout advancement. Reproducible osseous landmarks and sequential capsulolabral identification further enhanced procedural consistency. The small injection volume (1–2 mL) contributed to limiting unintended spread, allowing clear assessment of focal localization.

### 4.4. Role of Ultrasound Guidance and Feasibility of Targeted ALLC Injection

Ultrasound guidance enhances injection accuracy by enabling real-time visualization of both anatomical landmarks and needle trajectory, allowing precise targeting of the capsulolabral interface rather than diffuse intra-articular delivery.

In this cadaveric feasibility study, targeted injection to the ALLC at its glenoid attachment was consistently achieved, with injectate confined to the intended capsular–labral interface in both shoulders. These findings demonstrate that ultrasound-guided regenerative injections can reliably reach the ALLC under controlled conditions.

From a regenerative therapy perspective, accurate localization is critical, as therapeutic efficacy may depend on maintaining injectate concentration at the enthesis-level interface rather than allowing dispersion within the joint cavity. The present findings support the feasibility of interface-level delivery, which may improve the biological effectiveness of targeted interventions.

Several technical factors were identified as essential for successful delivery, including a cranial-to-caudal in-plane needle approach, continuous needle tip visualization, appropriate ultrasound frequency selection for deep tissue penetration, and the use of small-volume injectate to minimize unintended spread. Together, these factors contribute to reproducible and anatomically confined delivery to the ALLC.

### 4.5. Clinical Implications

Although this study did not evaluate biological outcomes, the anatomical precision demonstrated here establishes a foundational step for future clinical investigations. In younger patients with subtle anterior instability, localized compromise of the capsular–labral interface may contribute to symptomatic microinstability. In older patients, capsular thickening at the enthesis level may influence motion restriction.

Targeted regenerative delivery to the ALLC may therefore represent a biologically rational strategy to modulate localized pathology. However, clinical efficacy must be validated in prospective studies.

### 4.6. Limitations

This study has several limitations. First, it was conducted on a single fresh-frozen cadaver specimen. Although bilateral technical success was achieved, reproducibility across a larger and more diverse sample cannot be inferred.

Second, cadaveric tissue lacks physiologic perfusion, muscle tone, and inflammatory response, which may influence injectate distribution in vivo. Third, while red filler allowed gross visualization of confinement, it does not replicate the diffusion characteristics or tissue interactions of therapeutic agents such as prolotherapy solutions, PRP, or cell-based therapies.

Accordingly, this study was designed to provide proof-of-concept anatomical validation rather than establish clinical efficacy or statistical reliability.

Despite these limitations, this study demonstrates that ultrasound-guided injection can achieve precise and reproducible delivery to the ALLC at its glenoid attachment, with anatomically confirmed confinement of injectate at the capsulolabral interface.

These findings highlight the potential advantage of interface-level targeting over conventional intra-articular injection approaches, particularly in the context of regenerative therapies. This study provides a technical and anatomical foundation for future translational and clinical investigations.

## 5. Future Directions

Future investigations will include a larger series of cadaver specimens representing diverse anatomical characteristics to evaluate injectate diffusion patterns within and beyond the ALLC. Particular attention will be directed toward capsular confinement and potential communication with the joint cavity.

Subsequent clinical studies will be necessary to determine whether ALLC-targeted injection offers measurable benefit compared with conventional intra-articular approaches.

## Data Availability

The original contributions presented in this study are included in the article. Further inquiries can be directed to the corresponding authors.
